# Neurobiology of Hearing Loss and Ear Disease

**DOI:** 10.1155/2018/2464251

**Published:** 2018-04-19

**Authors:** Yong-Ho Park, Sung K. Moon, Kenneth H. Lee, Jeong-Han Lee

**Affiliations:** ^1^Department of Otolaryngology, College of Medicine, Chungnam National University, Daejeon, Republic of Korea; ^2^Department of Head and Neck Surgery, David Geffen School of Medicine, University of California, Los Angeles, Los Angeles, CA, USA; ^3^Department of Otolaryngology-Head and Neck Surgery, University of Texas Southwestern Medical Center, Dallas, TX, USA; ^4^Department of Physiology and Cell Biology, School of Medicine, University of Nevada, Reno, Reno, NV, USA

The interest towards research in hearing loss and ear disease has seen a great and progressive increase in the light of the continuous increase in the number of challenging research articles. Although hearing loss is a worldwide health problem and its socioeconomic burden is globally significant, nonregenerative hair cells in the cochlea and difficult drug delivery to the inner ear hindered progress in the field of hearing recovery [1]. However, with the advancement of molecular biology technologies and innovative imaging techniques, recent researches for hearing loss are focused on innovative approach such as gene therapy, stem cell therapy, and brain network analysis, heading one step forward to the new era of hearing research [2–4].

This special issue brings together 10 research articles and review article which nicely illustrate the wide spectrum of basic inner ear research and its related research about central auditory pathway. While some cutting-edge researches about neuroimaging study of central pathway including auditory cortex and higher cognitive function were illustrated, basic inner ear studies using many kinds of animal models were also described. In detail, diabetic mice, gerbil hearing loss model with aminoglycoside administration, and specifically devised early progressive hearing loss model were introduced. This special issue also includes deriving significant clinical implication, predicting the surgical outcomes from the data of cochlear implant recipients. Finally, the art of nanomedicine was thoroughly reviewed in the field of inner ear research.

The collection of papers published in this special issue nicely covers all the fields of hearing science including basic molecular study, imaging study, and clinical implication study and we hope that it will attract the interests of a large number of basic researchers as well as clinicians aiming at improving the treatment of hearing loss.

*Yong-Ho Park* *Sung K. Moon* *Kenneth H. Lee* *Jeong-Han Lee*
